# Pathogenic CD8+ T cells target K71+ Henle’s layer by forming cytolytic immune synapses in Alopecia Areata

**DOI:** 10.21203/rs.3.rs-9487026/v1

**Published:** 2026-04-30

**Authors:** Rupali Gund, Jin Y Kim, Valia P Leifer, Eunice Y Lee, Eddy HC Wang, Emily M. Mace, Angela M. Christiano

**Affiliations:** 1 Department of Dermatology, Columbia University Irving Medical Center, New York; 2 Department of Pediatrics, Columbia University Irving Medical Center, New York; 3 Department of Genetics and Development, Columbia University Irving Medical Center, New York; 4 Duncan and Nancy MacMillan Cancer Immunology and Metabolism Center of Excellence, Rutgers Cancer Institute of New Jersey, New Brunswick, NJ 08901, USA.

**Keywords:** Autoimmunity, CD8+ T cells, Cell-cell interactions, Hair loss, Henle’s Layer, Inflammation, Immune synapse, Skin Disease, T cell killing

## Abstract

CD8+ NKG2D+ T cells mediate hair follicle (HF) damage in Alopecia Areata (AA); however, the mechanisms and targets of T cell attack are not yet known. Here, we showed that CD8+ T cells penetrate the dermal sheath and epithelial layers of HF in AA and stop abruptly upon reaching Henle’s layer of the inner root sheath. CD8+ T cells assembled a cytolytic immune synapse (IS) with polarization of Lck kinase and convergence of granzyme B. The formation of IS and localization of caspase-8-dependent apoptosis in KRT71+ Henle’s layer of IRS pinpointed these cells as T cell targets in AA. Using direct visualization of *in situ* activity of pathogenic CD8+ T cells within AA-affected mice skin, we elucidated the molecular mechanism and target of CD8+ T cell cytotoxicity within HF in AA.

## Introduction

Alopecia areata (AA) is an autoimmune disease that leads to hair loss on the scalp or the entire body^1, 2^. We previously showed that CD8+ NKG2D+ T cells are the primary pathogenic T cells involved in AA^3, 4^. Research efforts in discovering new therapies for AA have been directed towards understanding the T cell-mediated recognition of HF targets and limiting T cell activation, migration, or inducing T cell exhaustion^4–8^. Despite significant progress made in identifying the phenotype and role of pathogenic T cells in AA, there are still gaps in understanding how T cells enter HF and the molecular mechanisms by which target cells are killed inside the HF. In many contexts, CD8+ T cells form cytolytic immune synapses (IS) that facilitate recognition and destruction of their target cells^9, 10^. Here, we investigated the mechanisms of HF damage by pathogenic T cells and the location of the target of pathogenic T cells within the HF in AA.

During classical IS formation, LFA-1 (expressed on T cells) binds to ICAM-1 (expressed on the antigen-presenting cell), mediating the initial T cell adhesion, which is followed by antigen-dependent recognition of the target cell^11,12^. Lck becomes phosphorylated and is polarized at the site of antigen engagement to initiate TCR signaling^13^. Upon target recognition, CD8+ T cells translocate the microtubule-organizing center (MTOC) towards the synaptic interface and polarize specialized secretory lysosomes containing soluble cytolytic proteins, such as granzymes, perforin, and the death-inducing Fas ligand^14^. MTOC docking ensures directional transport and polarization of lytic granules at the synaptic interface, allowing for the killing of target cells with high precision and without harming bystander cells^15^. Uptake of lytic granules by the target cell leads to caspase-mediated apoptosis and death of the target cell.

The hair bulb epithelium has been proposed as the primary target in AA based on the inflammatory cell infiltrate around the bulb region of anagen HFs^16^. It was hypothesized that the potential autoantigen in AA may be derived from melanocytes based on the clinical observation that pigmented hairs are lost preferentially to nonpigmented hairs in certain cases, and melanocytes are located in the bulb region of anagen HFs^17^. Identifying the specific HF cell populations that are selectively targeted and destroyed by pathogenic CD8+ T cells in AA may uncover novel therapeutic strategies for the disease.

Here, we defined the KRT71^+^ cells in Henle’s layer of the IRS as the major target of T cell damage. Using confocal microscopy, we elucidated that pathogenic T cells use immune synapses to kill target cells in the HF of AA-affected mice skin.

## Results

### CD8+ T cells penetrate across the dermal sheath to enter the HF in AA affected skin

**Figure 1.**
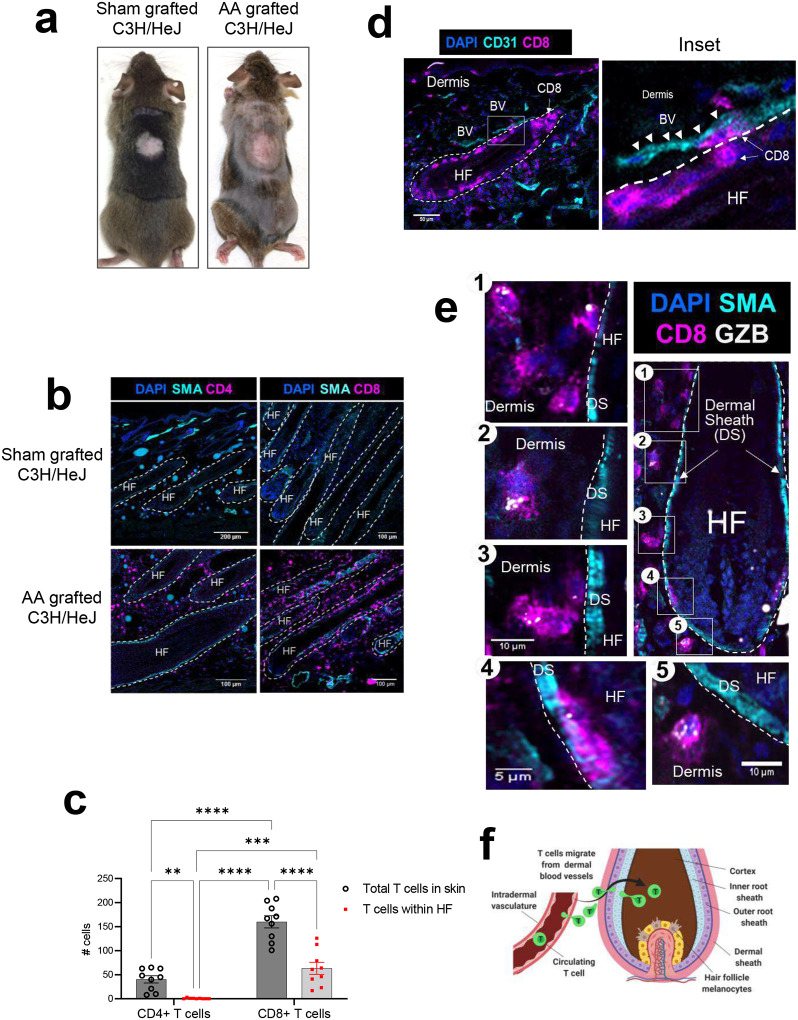
CD8+ T cells penetrate across the dermal sheath to enter the HF in AA affected skin while CD4+ T cells remain in the dermis. (a)Representative image of Sham- and AA grafted C3H/HeJ mice taken at 8 weeks after grafting. (b) Healthy and AA-affected skin from sham versus AA-grafted (6–9 weeks after grafting) stained for either CD4+ or CD8+ T cells (colored in magenta) along with labeling the dermal sheath with α-smooth muscle actin (SMA) staining (shown in cyan) to landmark the location of the HF. Nuclei were stained with DAPI dye shown in blue. White dashed line demarcates the location of the HF. (c) Quantification of number of CD4+ or CD8+ T cells located in the skin and within the HF. Data was combined by analyzing 3 AA mice and imaging 3 separate regions in the skin of each mice with each image containing 8–10 HF. Each data point on the graph represents cells counted in one microscopic field. Two-way ANOVA test was performed to determine statistical significance. **p<0.005, ***p<0.0005, and ****p<0.0001. (d) Skin from AA-grafted mice was stained with anti-CD31 antibody to label blood vasculature (BV shown in cyan, white arrow heads mark the location of BV close to HF) and CD8+ T cells located within skin and HF were also imaged (shown in magenta). Many CD8+ T cells were detected in either close proximity or within HF (White arrows). Nuclei were stained with DAPI dye shown in blue. White dashed line demarcates the location of the HF. Inset shows the magnified view of white box from d. White arrow points to CD8+ T cells captured exiting out of blood vessels (BV, white arrowheads) and entering HF. (e) CD8+ T cells (magenta) positive for granzyme B staining (grey) were imaged at different stages of migration into the HF that were demarcated with dermal sheath (DS) staining (cyan). Image 1–3 and 5 show T cells approaching the HF; Image 4 shows a T cell that has penetrated through the dermal sheath and is located inside the HF. White dashed line demarcates the location of the HF. (f) Diagram depicting a route for the T cells migration from the intradermal vasculature and across the dermal sheath to enter the HF.

To investigate the dynamic of pathogenic T cells in AA, we used the skin graft C3H/HeJ mouse model of AA (Fig.1a). In this model, lesional skin grafts harvested from AA-affected C3H/HeJ mice were transplanted onto unaffected C3H/HeJ recipients, resulting in nearly 100% of recipients developing AA hair loss within 6 weeks after grafting^4, 18^. Sham grafted mice, which did not develop AA, were used as controls. Using high-resolution confocal microscopy, we determined the spatial location and relative abundance of both CD4+ and CD8+ T cells in the skin and HF of Sham and AA grafted C3H/HeJ mice. We observed CD4+ and CD8+ T cells only in AA grafted C3H/HeJ, whereas no T cells were detected in Sham grafted C3H/HeJ (Fig. 1b). We found that while CD4+ T cells were mostly located in the dermis, numerous CD8+ T cells were also located within the HF in addition to being in the dermis and attached to the dermal sheath, (Fig. 1b & 1c), consistent with previous studies underscoring the role of CD8+ T cells in driving AA pathogenesis^4^.

Next, we investigated the mechanism by which CD8+ T cells entered the HF. We observed that many CD8+ T cells were in proximity to the intradermal blood vessels near the HF in AA grafted C3H/HeJ skin (Fig. 1d and inset). CD8+ T cells located outside the HF near the dermal sheath layer expressed granzyme B (GZB), suggesting that these cells were capable of cytotoxicity even before entering the HF (Fig. 1e). We detected GZB+ CD8+ T cells at various stages of entry into the HF. Some T cells were seen approaching or attached to the dermal sheath (Fig. 1e1, e2, e3 & e5), while other GZB+ CD8+ T cells were located inside the HF epithelial layers (Fig. 1e4). Based on these data, we determined that cytotoxic CD8+ T cells extravasated out of the intradermal blood vessels and penetrated through the dermal sheath to enter HF in AA-C3H skin (Fig. 1f).

### CD8+ T cells invade multiple epidermal layers within HF in AA grafted C3H/HeJ mice skin

We next sought to identify which layer of HF keratinocytes were targeted by CD8+ T cells in the skin of AA grafted C3H/HeJ mice. The HF is composed of several concentric cell layers: hair fiber at the center, the inner root sheath (IRS) that surrounds the hardened hair fiber, the companion layer, and the outer root sheath (ORS) that is contiguous with the interfollicular epidermis^19^. These layers exhibit transcriptionally distinct profiles, and coordinate their activity to produce the hair fiber^20^. We stained AA-C3H skin for CD8+ T cells and the following keratins proteins that specifically label keratinocytes of each layer – Keratin-14 (K14) for ORS, Keratin-75 (K75) for the companion layer, Keratin-71 (K71) for IRS, Keratin-82 (K82) for the cuticle, and AE13 for the hair cortex^20^.

**Figure 2.**
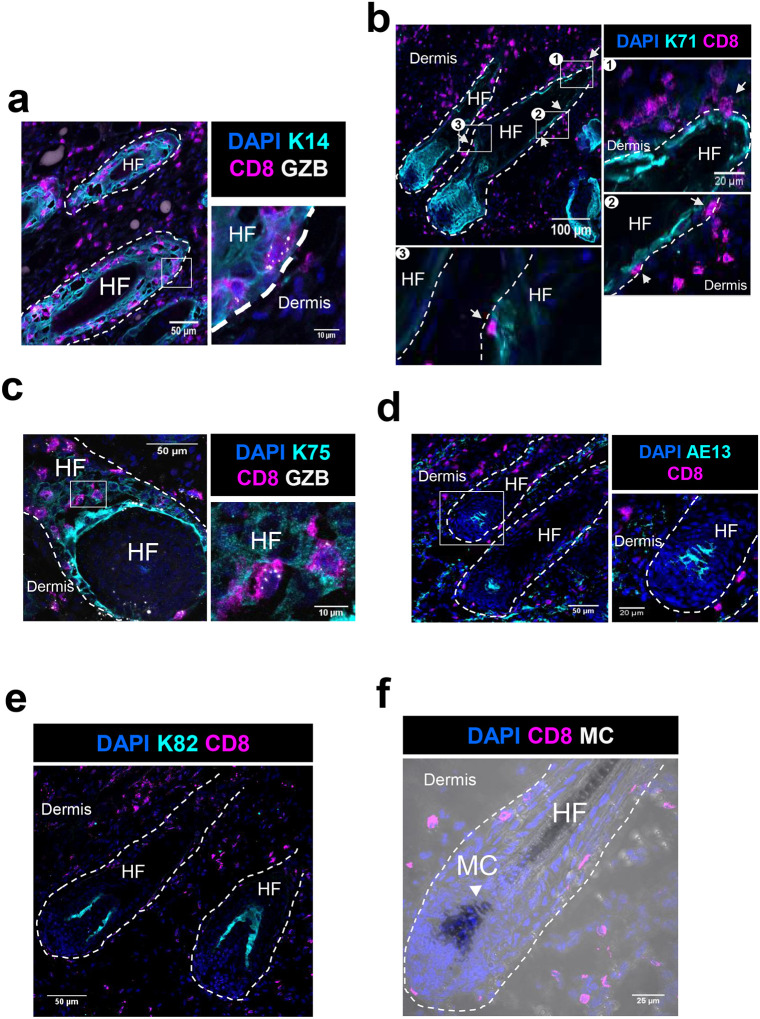
CD8+ T cells invade multiple epidermal layers within HF in AA affected skin. (a) Representative image showing CD8+ T cells (magenta) expressing granzyme B (GZB, grey) and located within Keratin 14 (K14, cyan) positive outer root sheath layer of the HF. Nuclei were stained with DAPI dye shown in blue. White box is magnified on the right to show GZB+ T cells located within K14+ layer of HF. White dashed line is drawn to distinguish hair follicles (HF) from dermis. (b) Representative images showing CD8+ T cells (magenta) contacting Keratin 71 (K71, cyan) positive inner root sheath layer of the HF. Nuclei were stained with DAPI dye shown in blue. White boxes are magnified to show CD8+ T cells (magenta, white arrows) contacting K71+ layer of HF. White dashed line is drawn to distinguish hair follicles (HF) from dermis. (c) Representative image showing CD8+ T cells (magenta) express granzyme B (GZB, grey) and located within Keratin 75 (K75, cyan) positive companion layer of the HF. Nuclei were stained with DAPI dye shown in blue. White box is magnified on the right to show GZB+ T cells located within K75+ layer of HF. White dashed line is drawn to distinguish hair follicles (HF) from dermis. (d) Representative image showing CD8+ T cells (magenta) located near AE13 (cyan) positive HF cortex. Nuclei were stained with DAPI dye shown in blue. White box is magnified on the right to show T cells located near hair cortex. White dashed line is drawn to distinguish hair follicles (HF) from dermis. (e) Representative image showing the location of CD8+ T cells (magenta) relative to Keratin 82 (K82, cyan) positive HF cuticle layer. Nuclei were stained with DAPI dye shown in blue. White dashed line is drawn to distinguish hair follicles (HF) from dermis. (f) Representative image showing the location of CD8+ T cells (magenta) relative to HF melanocytes (MC, detected by transmitted light imaging of dark melanin pigmentation in HF). Nuclei were stained with DAPI dye shown in blue. White dashed line is drawn to distinguish hair follicles (HF) from dermis.

We observed that CD8+ T cells had penetrated through K14, K75, and K71 expressing HF layers (Fig. 2a, 2b, and 2c respectively), but were not found in AE13 or K82 expressing epithelial layers (Fig. 2d, and 2E respectively). Importantly, higher magnification images revealed that CD8+ T cells located within the HF were GZB+ (Fig. 2a & 2c), suggesting that CD8+ T cells residing inside HF were activated to perform cytotoxic effector functions.

Melanocytes have been postulated to be the likely targets of pathogenic CD8+ T cells in AA due to preferential loss of pigmented hair^17, 21, 22^. Surprisingly, we detected CD8+ T cells only in proximity to, but not in direct contact with, HF melanocytes (Fig. 2f - representative images from data collected from 5 AA mice with at least 10 HFs analyzed from each mouse). Instead, we observed that CD8+ T cells invaded multiple HF epidermal layers and were found in proximity to different types of keratinocytes within HF. Strikingly, CD8+T cell invasion was abruptly arrested upon reaching the K71 expressing Henle’s layer of the IRS, suggesting that T cells encountered their cognate antigen(s) within this layer.

### CD8+ T cells assemble classical cytolytic immune synapses exclusively within HF in AA skin

**Figure 3.**
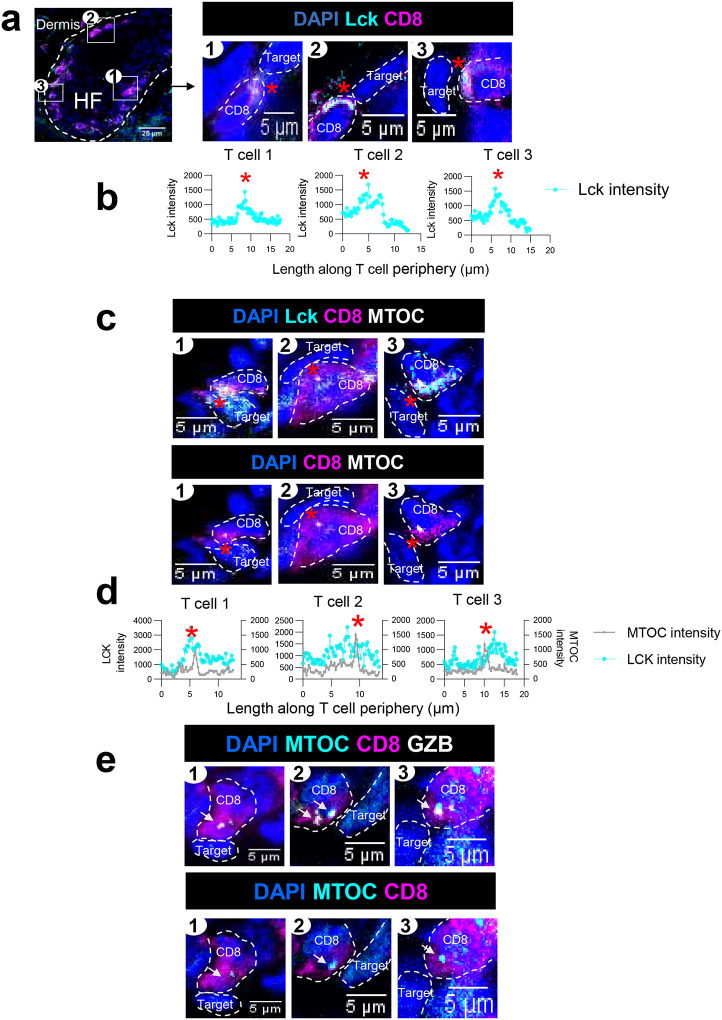
CD8+ T cells assemble classical cytolytic immune synapses within HF in AA skin. (a)Three representative HF resident CD8+ T cells (in magenta) are magnified on the right in panel 1, 2, and 3 to demonstrate Lck polarization (shown in cyan) at the site of T cell contact with neighbouring HF target cell (Red asterisks mark the contact site). Nuclei were stained with DAPI dye shown in blue. (b) Lck staining intensity along the boundary of T cells drawn based on CD8 staining. Lck staining is enriched at site of T cell contact with target cell region marked with red asterisks. (c) Three representative HF resident CD8+ T cells (in magenta) are shown in panel 1, 2, and 3 to demonstrate the location of MTOC (grey) and Lck polarization (cyan) at the T cell contact site (red asterisks) with the adjacent target HF cell. Nuclei were stained with DAPI dye shown in blue. (d) MTOC and Lck staining intensity along the boundary of T cells drawn based on CD8 staining. MTOC is located in the T cell where Lck staining is enriched at regions marked with red asterisks. (e) Three representative HF resident CD8+ T cells (magenta) are shown in panel 1. 2 and 3 to demonstrate the location of MTOC (cyan) and GZB (grey) at the contact site of T cell with the adjacent HF cell (white arrows). Nuclei were stained with DAPI dye shown in blue.

Next, we performed high-resolution imaging to probe the intracellular expression of molecules involved in target recognition and IS formation in HF invading CD8+ T cells. Lck is activated and polarized at the site of antigen binding that initiates the TCR signaling upon target recognition^23, 24^. We used total Lck staining to detect the site of antigen engagement in CD8+ T cells. In AA grafted C3H/HeJ skin, Lck expression in CD8+ T cells located inside the HF was polarized at the edge of T cells that were in contact with an adjacent HF cell (Fig. 3a, red asterisks mark the site of T cell contact with neighboring cell). Using ImageJ, we measured the Lck staining intensity along the T cell periphery that was defined by a line drawn along surface CD8 staining. Lck expression was enriched in the T cells at the site of T cell contact with the adjacent target cell (red asterisk in Fig. 3b), consistent with previous studies establishing the central role of Lck in initiating T cell signaling^25^. These data indicated that CD8+ T cells engaged and recognized target cells within the HF in AA grafted C3H/HeJ skin.

The docking of MTOC at the contact site with the target cell is a crucial event during assembly of the IS, which directs the lytic machinery to kill the target cell exclusively and prevent bystander killing^15^. We found that the MTOC was polarized and located close to the synaptic interface between CD8+ T cells and the adjacent HF target cell (red asterisks in Fig. 3c). Strikingly, the location of the MTOC overlapped with polarized Lck staining in the synapsing CD8+ T cells (red asterisks in Fig. 3c and Fig. 3d), indicating that T cell engagement was accompanied by MTOC translocation during IS assembly in pathogenic CD8+ T cells within the HF in AA grafted C3H/HeJ skin.

Cytotoxic CD8+ T cells utilize the IS to direct the secretion of granzyme B (GZB) containing lytic granules to specifically kill the target cell^10^. GZB-positive granules converge at the MTOC and translocate at the IS before being secreted into the synaptic cleft^15^. Therefore, MTOC polarization observed together with converged GZB is an indicator of T cell commitment to perform target cell killing^14^. We investigated whether MTOC polarization was linked with GZB convergence at the IS in HF invading CD8+ T cells in AA grafted C3H/HeJ skin. GZB was observed in proximity to the MTOC at the site of the CD8+ T cells contacting the HF target cell (white arrows in Fig. 3E). We detected various degrees of GZB convergence and polarization at MTOC in CD8+ T cells within the HF in AA grafted C3H/HeJ skin. Whereas some T cells had converged GZB that was situated very close to the MTOC (white arrows in Fig. 3e1 and 3e2), other T cells had converged GZB colocalized with the MTOC adjacent to the site engaging target HF cell (white arrow in Fig. 3e3). Taken together, these data demonstrated that pathogenic CD8+ T cells engage target cells within the HF and assembled a cytolytic IS to direct the lytic machinery toward the target cells in the HF of AA grafted C3H/HeJ skin.

We also measured the polarization of Lck and convergence of GZB at MTOC in CD8+ T cells located within the dermis, where CD8+ T cells are not in contact with their HF epithelial targets. In contrast to HF invading CD8+ T cells that showed Lck polarization at the site of contact with HF target cells (Fig. 3a), CD8+ T cells in the dermis had uniformly distributed Lck along the cell periphery (Supplementary Fig. 1a). Lck was not enriched at a certain edge of dermal CD8+ T cells as seen in HF invading CD8+ T cells (Supplementary Fig. 1b and Fig. 3b). We did observe GZB converging at or near the MTOC in dermal CD8+T cells (white arrows in Supplementary Fig. 1c), and there was no significant difference in the length of the shortest distance of GZB to the MTOC and number of GZB granules detected per cell between CD8+ T cells located in the dermis or within the HF (Supplementary Fig. 1d). These data suggest that T cells located within the dermis expressed cytolytic machinery but did not polarize Lck in the absence of encountering target cells.

### Cytotoxic T cells targets Krt71+ cells in Henle’s layer in AA

**Figure 4.**
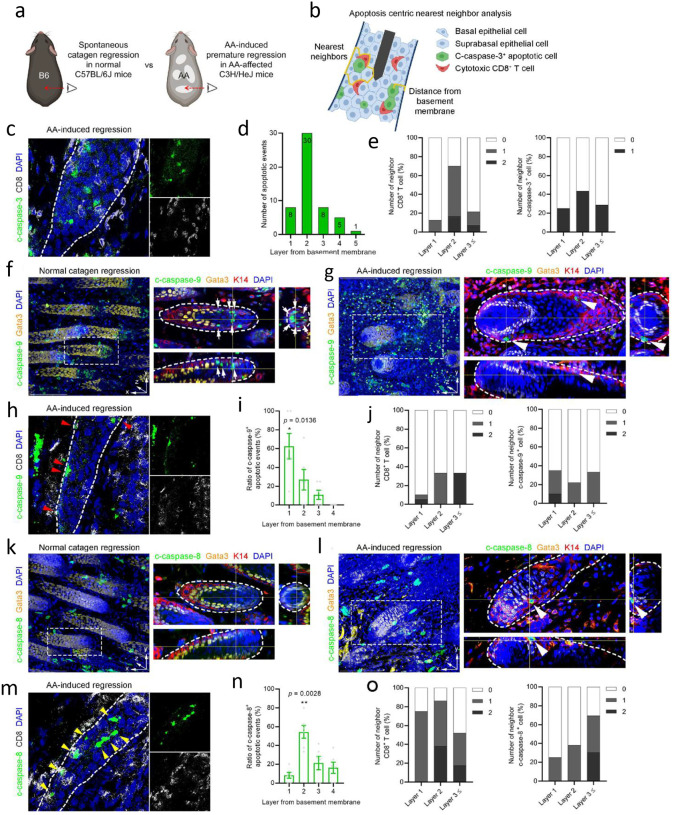
Caspase-8-dependent immune-mediated apoptosis defines premature HF regression in AA. (a) Comparison between the spontaneous HF regression in normal catagen of C57BL/6J mice and the premature HF regression in AA-affected C3H/HeJ mice. (b) Quantification strategy for apoptosis-centric nearest neighbor analysis. The number of c-caspase-3+ apoptotic cells was quantified by their distance from the basement membrane, along with the numbers of nearest neighboring CD8+ T cells and additional c-caspase-3+ cells. (c) Immunofluorescence of c-caspase-3^+^ dead cells and CD8^+^ T cells within AA-affected skin. (d) Quantification of immunofluorescence data in c showing the number of c-caspase-3+ dead cells within consecutive layers of cells from the basement membrane (*n* = 52 events from 3 mice), showing that most of the c-caspase-3^+^ apoptotic cells were found in the inner HF epithelial layers with one or more adjacent CD8^+^ T cells. (e) Quantification of immunofluorescence data in c showing the percentage of CD8+ T cell contacting a c-caspase-3^+^ dead cell within consecutive layers of cells from the basement membrane. (f) Whole-mount immunofluorescence of normal catagen regression, showing that many c-caspase-9^+^ cells were observed in both Gata3^+^ IRS layer and Krt14^+^ ORS layer (white arrows). (g) Whole-mount immunofluorescence of AA-induced regression showing that a few c-caspase-9^+^ cells were observed in Krt14^+^ ORS layer (white arrowheads). (h) Immunofluorescence of c-caspase-9^+^ cells within AA-affected skin. (i) Quantification of immunofluorescence data in h showing percentage of c-caspase-9^+^ cells within AA-affected skin. (j) Quantification of immunofluorescence data in h showing the numbers of nearest neighboring CD8^+^ T cells and adjacent another c-caspase-9^+^ cells by their distance from the basement membrane (*n* = 32 events from 3 mice) showing that a few of c-caspase-9^+^ cells were preferentially observed in the outer HF epithelial layer without adjacent CD8^+^ T cells. (k) Whole-mount immunofluorescence of normal catagen regression, showing that no c-caspase-8^+^ cells were detected. (l) Whole-mount immunofluorescence of AA-induced regression showing that a few c-caspase-8^+^ cells were observed selectively in the inner HF epithelial layers (white arrowheads). (m) Immunofluorescence of c-caspase-8^+^ cells within AA affected skin. (n) Quantification of immunofluorescence data in m showing the percentage of c-caspase-8^+^ cells within consecutive layers of cells from the basement membrane. (o) Quantification of immunofluorescence data in m showing the percentage of nearest neighboring CD8^+^ T cells and adjacent c-caspase-8^+^ cells by their distance from the basement membrane (*n* = 56 events from 3 mice), showing that most of the c-caspase-8^+^ cells were observed in the inner HF epithelial layers frequently with adjacent CD8^+^ T cells and other c-caspase-8^+^ cells. IRS, inner root sheath; ORS, outer root sheath. Data are mean ± s.e.m. *p < 0.05, **p < 0.01 (one-way analysis of variance with Dunnett’s multiple comparisons test).

To pinpoint the target cells of CD8+ T cell cytotoxicity in AA, we examined the early stages of disease using the lesional skin graft induced AA model in C3H/HeJ mice (Fig. 4a). While typical hair loss was observed around postgraft 6 weeks in AA-affected C3H/HeJ mice (Fig. S2B), perifollicular CD8+ T cell infiltration was detected with cleaved (c-) caspase-3+ apoptotic epithelial cells as early as postgraft 5 weeks (Fig. S2C). Notably, the timing of CD8+ T cell infiltration coincided with upregulation of MHC class I molecules on the HF ORS layer (Fig. S2C).

To determine the precise location of the target cells of T cell cytotoxicity in AA, we quantified the number of c-caspase-3+ apoptotic cells by their distance from the basement membrane and the numbers of nearest neighboring CD8+ T cells and additional c-caspase-3+ cells (Fig. 4b). We observed that most c-caspase-3+ apoptotic cells (Fig. 4c) were found in the inner HF epithelial layers (Fig. 4d) with one or more adjacent CD8+ T cells (Fig. 4e). Caspase-3 is the final effector in the apoptotic cascade that converges from both caspase-9-dependent intrinsic pathway (spontaneous apoptosis) and caspase-8-dependent extrinsic pathway (immune-mediated apoptosis)^26^.

To test whether any of the apoptotic epithelial cells specifically underwent caspase-8-dependent immune-mediated apoptosis due to CD8+ T cell-mediated cytotoxicity, we examined c-caspase-9+ cells and c-caspase-8+ cells during catagen regression in wildtype C57Bl/6 mice and premature regression in AA grafted C3H/HeJ mice. Mice from C57Bl/6 strain were used as control for this experiment, as the C57Bl/6 strain does not spontaneously develop alopecia with age. In normal regression, several c-caspase-9+ cells were observed in both Gata3+ IRS layer and Krt14+ ORS layer (Fig. 4f). In C57Bl/6 catagen skin, only a few c-caspase-9+ cells were observed in Krt14+ ORS layer in AA-induced regression in C3H/HeJ mice (Fig. 4g). Quantification showed that a few of the c-caspase-9+ cells (Fig. 4h) were preferentially observed in the outer HF epithelial layer (Fig. 4i) without adjacent CD8+ T cells (Fig. 4j) in AA-affected C3H/HeJ mice.

Notably, no c-caspase-8+ cells were detected during normal catagen regression (Fig. 4k), whereas c-caspase-8+ cells were observed selectively in the inner HF epithelial layers in AA-induced regression in C3H/HeJ mice (Fig. 4L). Quantification showed that most of the c-caspase-8+ cells (Fig. 4m) were observed in the inner HF epithelial layers (Fig. 4n), frequently with adjacent CD8+ T cells and other c-caspase-8+ cells (Fig. 4o) in AA-affected C3H/HeJ mice. These data suggest that CD8+ T cells induce an immune-mediated c-caspase-8-dependent extrinsic cell death pathway in damaged HF in AA.

### CD8+ T cells IS formation coincides with the killing of cells within Henle’s layer of HF inner root sheath

**Figure 5.**
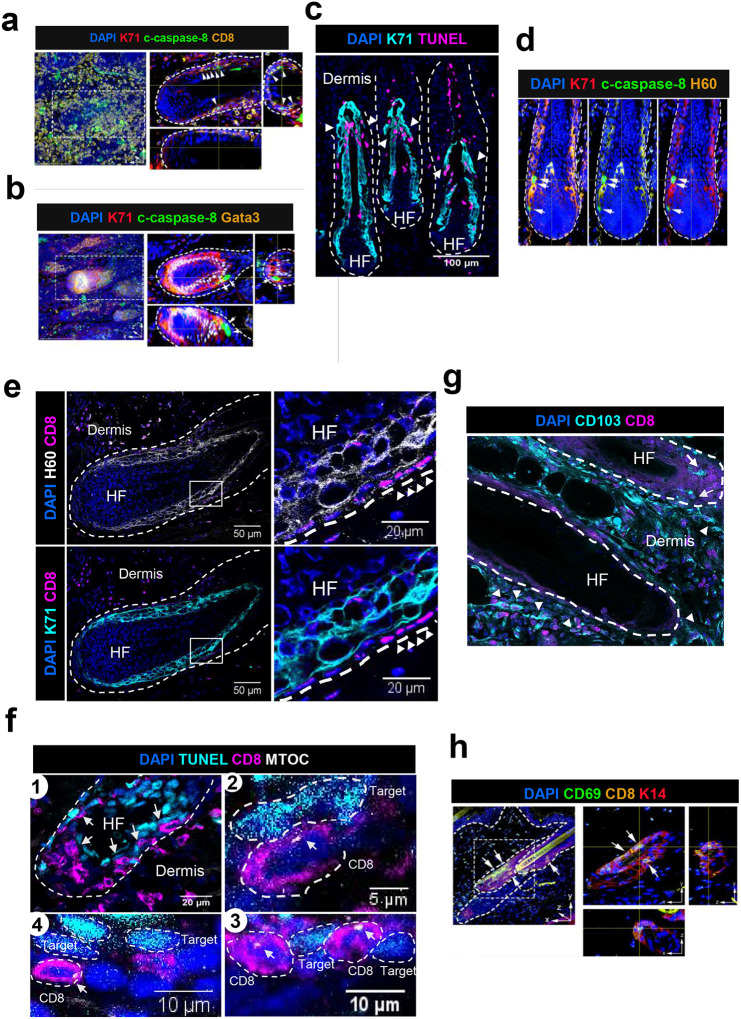
CD8+ T cells immune synapse formation coincides with the killing of K71+cells within Henle’s layer of HF inner root sheath. (a) Whole-mount immunofluorescence of AA-induced regression showing that several c-caspase-8^+^ cells observed selectively in Krt71^+^ IRS layer (white arrowheads) with adjacent CD8^+^ T cells. (b) Whole-mount immunofluorescence of AA-induced regression showing that most of c-caspase-8^+^ cells were primarily located within the Krt71^+^Gata3^−^ IRS layer (white arrows), which is defined as Henle’s layer, the outermost layer among the three distinct IRS layers of anagen HFs. (c) Many of the TUNEL+ dead cells (in magenta) are located within K71+ (cyan) Henle’s layer of inner root sheath cells (white arrowheads). Nuclei were stained with DAPI dye shown in blue. The white dashed line demarcates the region of HF. (d) Immunofluorescence of AA-affected C3H/HeJ mice showing that the Krt71+ IRS layer expressing H60 (white arrows), a ligand for the NKG2D receptor, contains c-caspase-8+ dead cells. (e) CD8+ T cells (in magenta) colocalize near K71+ (cyan) Henle’s layer of inner root sheath cells that also express NKG2D ligand H60 (grey). Nuclei were stained with DAPI dye, shown in blue. The white dashed line demarcates the region of HF. White box is magnified on the right to show the location of CD8+ T cells (white arrowheads) in proximity to H60-expressing K71 HF cells. (f) Panel 1 shows the low magnification image of CD8+ T cells (in magenta, white arrows) surrounding TUNEL+ dead cells (cyan) within HF of AA-affected C3H/HeJ mice skin. Panel 2–4 shows three representative images of CD8+ T cells (magenta) located in proximity of TUNEL+ dead cell (cyan). CD8+ T cell (magenta) in panel 2 is observed to polarize MTOC (grey) at the site of contact (white arrow) with dead TUNEL+ target cell (cyan). (g) CD8+ T cells (magenta) double-positive for CD103 (cyan) labeling Trm cells were found within HF (white arrows) and in the dermis near HF (white arrowheads) in AA-affected C3H/HeJ mice skin. Nuclei were stained with DAPI dye shown in blue. White dashed line demarcates the region of HF. (h) Whole-mount immunofluorescence showing CD8+ T cells in the upper regions of HF which expressing tissue-resident memory T cell marker CD69.

We observed c-caspase-8+ cells selectively in the Krt71+ IRS layer with adjacent CD8+ T cells using whole-mount staining (Fig. 5a). Most of c-caspase-8+ cells were located within the Krt71+Gata3− IRS layer (Fig. 5b), which is defined as Henle’s layer, the outermost layer of the three distinct IRS layers of anagen HF^27^. These findings indicated that premature HF regression in AA is defined by caspase-8-dependent immune-mediated apoptosis in the Krt71+ Henle’s layer. Notably, we detected both TUNEL+ and c-caspase-8+ apoptotic cells exclusively within the K71+ Henle’s layer, which corroborates that Krt71+ cells in Henle’s layer were specifically targeted by pathogenic CD8+ T cells in AA (Fig. 5c and 5d, white arrows point to location of dead cells within K71+ Henle’s layer).

We previously showed that the Henle’s layer of the IRS also expresses H60 and Rae-1, which are ligands for NKG2D receptor that is expressed on alopecic CD8+ T cells^4^. Here, we observed that CD8+ T cells were located adjacent to and in direct contact with H60-expressing K71+ cells within Henle’s layer (Fig. 5e, white arrowheads point to location of CD8+ T cells within HF). To further confirm whether CD8+ T cell immune synapse formation with Krt71+ IRS layer resulted in the killing of target cells in Henle’s layer, we performed dual staining for TUNEL+ dead cells and MTOC to directly image the location of the immune synapse relative to the dead target cells within the HF of AA grafted C3H/HeJ skin. We detected numerous TUNEL+ cells surrounded by a ring of CD8+ T cells within the HF (Fig. 5f1). We found polarization of MTOC in the CD8+ T cells that were located adjacent to or in direct contact with the dead TUNEL+ cells (Fig. 5f2–4, white arrow points to the MTOC). Polarization of MTOC in the CD8+ T cells within HF suggests that T cells were redirecting their cytolytic machinery towards the target cells causing cell death. Together, these data demonstrate that T cell cytolytic immune synapse formation coincided with the killing of the target cell, specifically within K71+ cells in Henle’s layer of the IRS. Further, we investigated the phenotype of CD8+ T cells located within AA skin and found CD103, a marker for tissue resident memory T cells (Trm), was upregulated in pathogenic T cells found near the HF (Fig. 5g). Interestingly, we also found CD8+ T cells within HFs expressing another Trm marker CD69 (Fig. 5h). Proximity of Trm cells to HF in AA skin suggests that Trm may be generated in response to presumptive AA autoantigens expressed within HF.

### KRT71+ Henle’s layer harbors intrinsic immunogenicity and is damaged in AA patients

**Figure 6.**
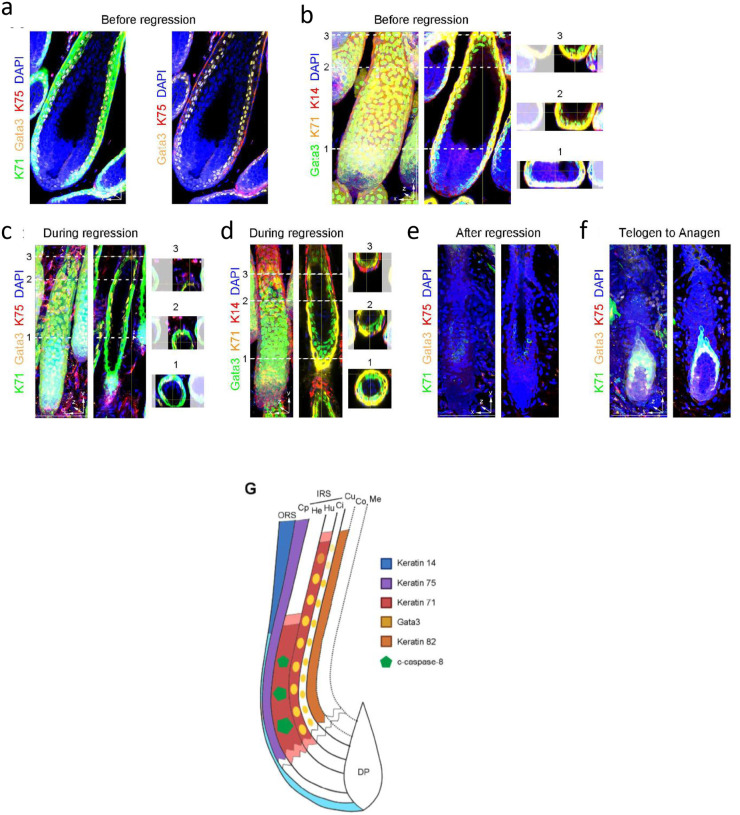
Spatiotemporal distribution of Krt71+ Henle’s layer defines the extent of HF damage (a) and (b) Whole-mount immunofluorescence showing that Krt71+ IRS cells was located in-between Gata3+Krt71−IRS layer and Krt75+ companion layer, which is surround by very thin Krt14+ ORS layer, in the proximal bulb region of anagen HFs before regression.(c) and (d) Whole-mount immunofluorescence showing that a transition zone exhibiting an abrupt cessation of Krt71 and Gata3 expression in the IRS layer, where Krt14 expression became prominent in the ORS layer, in the supra-bulb region. (e) and (f) Whole-mount immunofluorescence showing Krt71+ IRS cells were no longer observed in the bulge region of telogen HFs after regression, and Krt71+ IRS cells reappeared upon the initiation of the subsequent anagen. (g) Schematic illustration of the transition zone between the bulb region and the supra-bulb region, which restricts the Krt71+ IRS cells and corresponding T cell-mediated cytotoxicity within the proximal bulb region of anagen HFs.

To determine whether the same findings are recapitulated in human AA patients, we examined human HFs of normal control scalp and non-lesional and lesional scalp in AA patients. In normal control scalp, no c-caspase-8+ cells were detected in the HF epithelium (Fig. 6a). In contrast, we found that c-caspase-8+ cells were primarily located within the KRT71+ IRS layer in lesional scalp of AA patients (Fig. 6b), but not in the KRT14+ ORS layer or in the hair matrix region, consistent with the findings in AA grafted C3H/HeJ mice. The KRT71+ IRS layer in lesional scalp showed high expression of ULBP2, ULBP5, and ULBP6 (Fig. 6c), ligands for the NKG2D receptor in humans^2, 4^. Recently, our whole-exome sequencing (WES) analyses on 849 AA patients revealed that damaging KRT82 mutations are rare variants associated with AA, in conjunction with reduced KRT82 expression^28^. The KRT71+ IRS cells were located adjacent to the KRT82+ cuticle layer, and decreased KRT82 expression was observed along with decreased KRT71 expression in damaged HFs of the lesional scalp (Fig. 6d), suggesting that disruption in the KRT82+ cuticle layer may interact with the KRT71+ IRS cells harboring potential self-antigens in AA.

### NKG2D ligand expression is upregulated in Henle’s layer of the inner root sheath in AA

**Figure 7.**
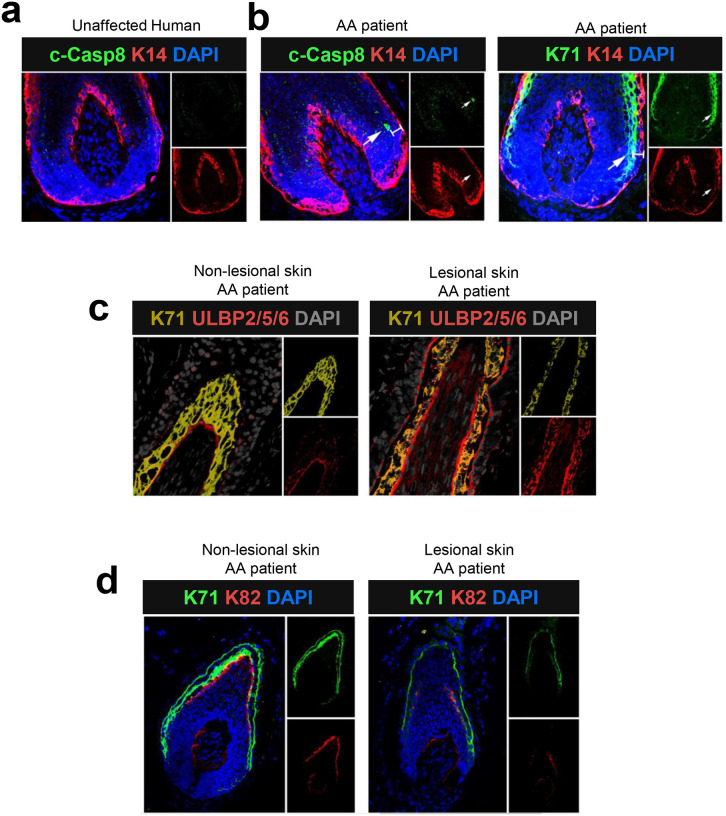
KRT71+ Henle’s layer harbors intrinsic immunogenicity and is damaged in AA patients (a) and (b) Immunofluorescence showing that c-caspase-8+ cells (white arrows) were primarily located within the KRT71+ IRS cells in lesional scalp of AA patients, whereas no c-caspase-8+ cells were observed in normal control scalp. (c) Immunofluorescence of human AA patients showing that the KRT71+ IRS layer in lesional scalp showed high expression of ULBP2/5/6 ligands for the NKG2D receptor in humans. (d) Immunofluorescence of human AA patients showing the KRT71+ IRS cells was located just outside the KRT82+ cuticle layer and decreased KRT82 expression was observed along with decreased KRT71 expression in damaging HFs of lesional scalp.

To determine whether cytotoxic T cells target specific cellular component(s) of the hair follicle in AA, we performed single cell RNA-sequencing (scRNAseq) on skin samples from ungrafted and grafted C3H/HeJ mice. We evaluated expression of all murine NKG2D ligands (*Ulbp1, Ulbp2, Ulbp3, Mica, Micb, H60a, H60b, H60c, Rae1, Mult1*) in dermal mesenchymal cell populations, and observed expression of *Rae1, Ulbp1, and H60c* (Figure 7). We then performed differential gene expression analysis using Wilcoxon rank sum testing to determine whether visual differences in gene expression among dermal mesenchymal cell populations seen in violin plots were statistically significant (p<0.05). We found that Henle’s layer of the inner root sheath (IRS) displayed increased expression of *Rae1* and *H60c* in AA mice compared to ungrafted (UG) mice, and that a mixed cluster of IRS cells (“IRS”) displayed increased expression of H60c in AA mice compared to UG mice (supplementary tables 2–4). We classified the IRS cluster as mixed, as it contained markers attributable to all three inner root sheath cell layers (Huxley, Henle, IRS cuticle).

**Figure 8.**
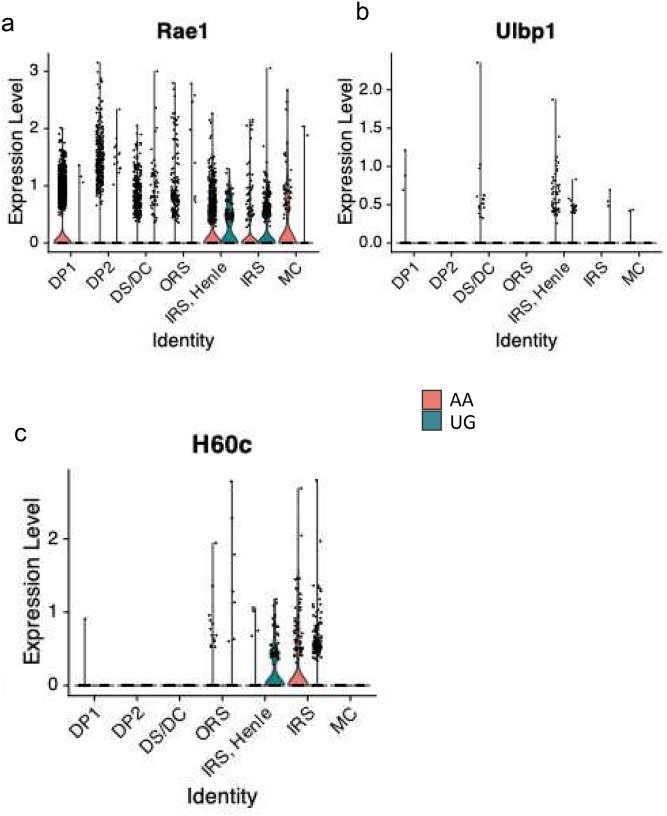
Violin plots depicting normalized and scaled NKG2D ligand scRNAseq expression data in AA and UG dermal mesenchymal cell populations. Gene expression for a given cluster for AA and UG are shown in pink and blue, respectively. (a) Rae1 expression by cell cluster. (b) Ulbp1 expression by cell cluster. (c) H60c expression by cell cluster. AA, alopecia areata grafted C3H/HeJ mice; UG, ungrafted C3H/HeJ mice; DP1/2, dermal papillae clusters 1/2; DS/DC, dermal sheath/dermal cup cells; ORS, outer root sheath cells; IRS, Henle, Henle’s layer of inner root sheath cells; IRS, inner root sheath cells; MC, melanocytes.

*Rae1* expression was increased in Henle’s layer in AA by a log2 fold-change (log2FC) of 0.24 compared to all AA and UG dermal cell clusters (supplemental table 2). Of note, AA dermal papillae cell cluster 1 also displayed statistically significant upregulation of *Rae1* (supplemental table 2). The apparent visual increase in *Rae1* expression in UG IRS cells compared to AA IRS cells in Figure 8a did not emerge as statistically significant. While *Ulbp1* was increased in Henle’s layer in both AA and UG mice compared to all other dermal cell clusters, the log2FC was significantly higher in Henle’s layer in AA compared to UG (3.78 vs. 2.85; supplemental table 3). *H60c* exhibited a similar pattern of increased expression in the IRS cluster in both AA and UG, with a higher log2FC in AA compared to UG (3.90 vs. 3.47; supplemental table 4). Lastly, of note, melanocytes did not exhibit statistically significant increases in NKG2D expression in any AA cell population.

## Discussion

We report the *in situ* interactions of pathogenic CD8+ T cell with HF target cell in C3H/HeJ mouse model of AA. Our studies revealed that CD8+ T cells traversed multiple epidermal layers and established an immune synapse upon reaching K71 expressing Henle’s layer of the IRS within HF. Synapsing CD8+ T cells within the HF showed strongly polarized Lck and GZB close to the MTOC at the target contact site, providing evidence of direct engagement with target cells. CD8+ T cells remained in contact with dead TUNEL+ and c-caspase-8+ cells located within K71+ Henle’s layer of the IRS, demonstrating that T cells killed the epithelial cells in the Henle’s layer to induce HF damage in AA.

Our findings are consistent with previous studies that found T cells targeting keratins K71 and K31 (expressed in Henle’s layer and the hair cortex respectively) were expanded in AA affected C3H/HeJ mice, and further, that vaccination with K71 and K31 derived peptides was sufficient to induce AA in C3H/HeJ mice^29^, suggesting that K71 is perhaps a bona fide AA autoantigen. Our whole exome sequencing in AA patients discovered predisposing genetic variants in the KRT82 that is expressed adjacent to Henle’s layer in the hair cuticle in human patients with AA^28^. These findings invite renewed investigation into keratins as AA autoantigen, particularly in light of the previous reports of keratin proteins as potential AA autoantigens in both humans and C3H/HeJ mice affected with AA^30, 31^.

Henle’s layer of the IRS may also play a role in the collapse of immune privilege in the HF during AA. Notably, we previously observed that the NKG2D receptor ligand, H60, was upregulated in AA HFs, and was expressed specifically in Henle’s layer^4^. We previously reported that H60 was expressed at higher levels at baseline in C3H/HeJ mice compared to B6 mice, suggesting that elevated expression of H60 may predispose to autoimmunity against the HF^4^. Along these lines, it was previously reported that transgenic ectopic expression of RAE1ε (a murine NKG2DL) in the pancreas induced the active recruitment of cytotoxic CD8+ T cells and led to the development of insulitis, suggesting that NKG2D ligation can break peripheral tolerance and promote responses to altered self in both physiological immunosurveillance and autoimmune disease states^32^. Thus, H60 overexpression within the K71+target layer undergoing CD8+ T cell attack in AA suggests that overexpression of H60 in Henle’s layer may induce the recruitment of cytotoxic CD8+ T cells to the HF and contributes to the breakdown of immune privilege in AA.

It is well-established in the literature that CD8+ T cells in AA preferentially induce damage in actively growing anagen hair follicles, which results in accelerated entry into premature catagen phase^16, 33^. This observation led to the speculation that proteins exclusively expressed in anagen hair follicles may be the source of antigen for AA. The clinical observation of sudden loss of pigmented hair causing overnight hair greying led to the hypothesis that HF melanocytes may be the source of antigens that are targeted by T cells^17, 21, 22^. Although previous reports have detected melanocyte antigens with AA in both patients and C3H/HeJ mice affected with AA, in this study, we did not detect CD8+ T cells in direct contact with HF melanocytes. Although CD8+ T cells were found in proximity of melanocytes, we did not observe TUNEL+ cells in the region corresponding to the HF melanocytes within hair bulb even at later stages of disease in AA affected C3H/HeJ mice with more than 70% body hair loss (Fig. 2f & Fig. 5b), suggesting that CD8+ T cells do not target melanocytes in this model. In addition, from our single cell data from whole skin of C3H/HeJ mice affected with AA, we found that melanocytes also do not express NKG2D ligands. HF melanocytes are embedded deep within the hair bulb, requiring that T cells would need to penetrate many surrounding HF epithelial layers before they could access the HF melanocytes. In light of these findings, it appears unlikely that melanocytes are the target cells for CD8+ T cell mediated killing in the C3H/HeJ model of AA.

Our work uncovered a novel mechanism and defined the key molecular events involved in CD8+ T cell IS formation in AA. We determined the *in situ* behavior of pathogenic T cells within the skin of AA grafted C3H/HeJ mice from their entry into HF and T cell-mediated killing of Henle’s layer within HF. Taken together, these findings define the mechanism of CD8+ T cell-mediated cytolytic immune synapse formation in HF damage and advance our understanding of classical autoimmune mechanisms in AA. Interestingly, there are several case reports of AA patients improving after extended treatment with efalizumab, an anti-CD11a subunit of LFA-1 that inhibits T cell adhesion and prevents immune synapse formation^34, 35^, however a lack of efficacy was seen in a clinical trial of short-term duration, and this drug was later withdrawn from the market)^36^. Our findings invite new potential therapeutic approaches aimed at blocking surface molecules such as LFA-1 and/or ICAM-1 for the treatment of AA, perhaps using local delivery of small molecule inhibitors such as Lifitegrast to block IS formation^37^.

Our study revealed the presence of Trm near HF in AA skin is consistent with the published clinical report that lesional AA skin contains significantly more CD103+CD8+ T cells as compared to non-lesional skin^34^. Conventionally, Trm are generated in response to skin viral infections and persist at the site of infection to confer long-term protection^35–37^. The close proximity of Trm to HF in AA skin suggests that Trm may be generated in response to presumptive AA autoantigens. Given the behavior of Trm to persist in target tissue niche and in close proximity to the HF, Trm may participate AA relapse after withdrawal of T-cell targeted therapies such as JAK inhibitors once the hair attempts to re-enter the hair cycle into anagen, possibly due to reappearance of putative autoantigens in the skin instigating Trm reactivation^38^. Targeting the IS could also comprise a combination treatment strategy after HF has recovered in response to a JAK inhibitor, to protect the newly grown hairs from subsequent attack by Trm by blocking new IS formation. Targeting the Trm themselves could also provide durable responses to treatment by eliminating these recalcitrant T cells altogether from their niche.

## Materials and Methods

### Mice

8–9 weeks old C3H/HeJ female mice were purchased from the Jackson Laboratory and kept under specific pathogen-free conditions in the small animal facility at the Columbia University Irving Medical Center. All experiments were performed in compliance with guidelines as approved by the Institutional Animal Care and Use Committee of Columbia University Irving Medical Center.

### Antibodies and reagents

Antibodies used in the study are provided as a Supplementary table 1.

Tissue-Plus^™^ O.C.T. Compound Tissue-Plus^™^ O.C.T. Compound (Fischer Scientific), BD cytofix/cytoperm (Cat. No. 554722), Fluoroshield^™^ with DAPI histology mounting medium (Sigma-Aldrich, F6057-20ml), Click-iT^™^ Plus TUNEL Assay for In Situ Apoptosis Detection, Alexa Fluor^™^ 594 dye (Invitrogen^™^ C10618), Microscope Slides (Fisher Scientific, 12-550-15), MOM Block (Vector laboratories, BMK-2202), Normal Donkey serum (Jackson ImmunoResearch, 017-000-121), Immedge^™^ Hydrophobic Barrier Pen (Vector Laboratories, H-4000).

### Experimental induction of Alopecia Areata

10-weeks old female recipient C3H/HeJ mice were grafted with skin isolated from chronic AA donor mice. Mice were monitored for wound healing for two weeks after which the sutures were removed. Recipient mice developed AA disease within 6–8 weeks after grafting. Skin biopsies were collected from mice that develop disease at 6–9 weeks after grafting and processes for microscopic analysis of immune cells.

### Skin processing, embedding, sectioning and Immunofluorescent staining

Skin biopsies collected from AA mice were embedded in Tissue-tek OCT solution and kept frozen at −80° C until use. 5–10 μm thick sections were cut and collected onto the microscopic slides. Frozen skin section was fixed using BD cytofix/cytoperm buffer for 10 minutes in a humidifying chamber at ambient temperature and slides were soaked in PBS twice for 5 min each. Hydrophobic barrier was drawn around the skin section using Immedge pen and then skin section was incubated with blocking buffer (containing 5% normal donkey serum+ 5% M.O.M. Mouse Ig Blocking Reagent in 0.1%Triton-X 100/PBS) for 1 hour at ambient temperature.

Slides were then soaked in TBS buffer containing 0.1% Tween-20 twice for 5 mins each and incubated with antibody dilution buffer (containing 8% M.O.M. Protein Concentrate in 0.1% Triton-X 100/PBS) for 5 min at ambient temperature. The antibody dilution buffer was then removed by vacuum aspiration. The skin section was incubated with primary antibodies (diluted 1:100 in antibody dilution buffer) overnight at 4° C.

Next day, slides were soaked in TBS buffer containing 0.1% Tween-20 three times for 5 mins each to remove unbound primary antibodies and incubated with secondary antibodies (diluted 1:250 in 5% normal donkey serum +0.1% Triton-X 100/PBS) for 30 mins at ambient temperature. Slides were then soaked in TBS buffer containing 0.1% Tween-20 three times for 5 mins each to remove unbound secondary antibodies, mounted in Fluoroshield with DAPI liquid and stored at 4°C in dark for imaging next day.

### Whole-mount immunofluorescence staining

For whole-mount immunofluorescence on pieces of intact tissues, back skins were harvested and cut into 1 cm × 0.8 cm strips fixed in 4% paraformaldehyde/PBS at 4°C overnight before peeling away the panniculus carnosus muscle. Subsequent steps were then performed in a 24-well plate. Skins were then treated with a 0.3% Triton X-100, 5% donkey serum, 20% DMSO/PBS solution for 8 h before primary antibody labeling against c-caspase-9 (1:200), c-caspase-8 (mouse, 1:200), Gata3 (1:100), Krt14 (1:500), CD8 (mouse, 1:100), Krt71 (1:100), Krt75 (1:100), c-caspase-3 (1:400), CD69 (1:100), CD103 (1:100) in the Triton X-100/donkey serum/DMSO/PBS solution at 25°C for 5 days with gentle shaking. After washing in 0.3% Triton X-100/PBS solution at 25°C for 8 h with buffer changes every 30 min, tissues were then stained with donkey or goat anti-rabbit, rat, chicken, mouse, goat, or guinea pig antibodies conjugated with AlexaFluor 488, 594 or 647 (Invitrogen) in the Triton X-100/donkey serum/DMSO/PBS solution used similarly with primary antibody labeling at 25°C for 3 days with gentle shaking. Finally, tissues were washed again in 0.3% Triton X-100/PBS solution at 25°C for 8 h with buffer changes every 30 min and counter stained with DAPI. Optical clearing was then performed by dehydrating tissue in 50:50 methanol/water for 5 min then a series of three 100% methanol treatments at 25°C for 30 min. Final clearing was performed in a BABB (benzyl alcohol/benzyl benzoate, 1:2 ratio, Sigma) solution until visibly clear then mounted in residual BABB held in a custom chambered glass slide. Antibodies used in this study are listed in the Supplemental Table 1.

### Image acquisition and analysis

Immunofluorescence-stained skin sections were imaged with either Nikon Ti Eclipse inverted microscope with A1 scanning confocal unit or Leica Dmi8 with Stellaris 5 confocal microscope system equipped with Leica LAS X software. Z-stacks were acquired using 20X dry or 60X oil objective (with a minimum step size of 0.5 μm). Image stacks were analyzed and adjusted for brightness and contrast to create Fig. panel using ImageJ/FIJI (NIH)^38^. Multi-color image was split into separate channel. Staining for CD8 coreceptor was used to landmark T cell surface and Lck expression at the synapse was measured by assessing Lck fluorescence along a line drawn on the T cell surface staining positive for CD8. Length of the shortest distance of GZB+ granules to MTOC and number of granules per T cells were measured using spots model in Imaris 9.8 software.

### Statistical Analysis

GraphPad prism was used to analyze data comparing two or more groups with two-tailed unpaired Student’s t-test or two-way ANOVA respectively. All data are presented as mean ± SEM. **p<0.005, ***p<0.0005, and ****p<0.0001.

#### Mouse Skin Preparation of Single-cell Suspensions

After euthanasia in accordance with Columbia University’s IACUC guidelines, mice were shaved using a clipper prior to harvesting the dorsal skin. Subcutaneous adipose tissue was then harvested, and skin was floated on 0.25% trypsin (Thermo Fisher Scientific, #15090046; diluted 10X in PBS) for one hour at 37°C with the dermis side down. After neutralization of trypsin with DMEM + 10% FBS, the epidermis was separated from the dermis using a scalpel. The epidermis was filtered through a 70μm strainer and centrifuged at 300g, 40°C, then resuspended in DMEM +10% FBS. The dermis was cut into small pieces for digestion using 0.25% collagenase type IV (Sigma-Aldrich, #C5138) in DMEM at 37°C for one hour in a shaking incubator. Collagenase was then neutralized using DMEM + 10% FBS and digested skin was passed through a 70μm strainer, from which the flow through was rinsed twice via centrifugation at 300g, 40°C.

### FACS for single-cell RNA-sequencing

Suspensions from the epidermis and dermis were resuspended and washed in 2% FBS in Cell Staining Buffer (BioLegend, #420201). Cells were strained through a 50μm filter, and then samples were stained with DRAQ5 (Biolegend #424201; 1:1000) and SYTOX-Green (Thermo Fisher Scientific, #S7020; 1:500). Live DRAQ5-SYTOX-Green-cells were then isolated for single-cell RNA-sequencing (scRNAseq).

### Single cell 3’ mRNA Sequencing

All scRNAseq samples were sent to the JP Sulzberger Columbia Genome Single Cell Analysis Core for processing. 10X Genomics Chromium Single Cell 3’ Reagent Kits were used to prepare libraries according to manufacturer instructions, targeting 5,000 cells and 350 million reads. An Illumina NextSeq 500/550 was used to sequence libraries.

### Processing and analysis of scRNAseq data

The Cell Ranger pipeline (v3.0.1 and mm10 reference genome for mouse scRNAaseq experiments, v3.0.2) was used to align FASTQ files. Cell Ranger output files (barcodes.tsv, genes.tsv, and matrix.mtx) for each sample were loaded into R (v4.3.2), where cells were processed for quality control using Seurat (v5.1.0)^39^ to exclude low quality cells and doublets with <200 unique molecular identifier (UMI) counts, over >5,000 UMI counts, or >15% mitochondrial RNA, and to exclude features detected in ≤3 cells. For this analysis, we focused on samples from the dermis of ungrafted (control) and grafted (AA-affected) samples. After quality control and processing, ungrafted and grafted samples yielded n=14,262 and n=3,516 cells, respectively.

Separate Seurat objects were created for ungrafted (control) and grafted (AA-affected) samples, and processed using the following steps with default Seurat parameters before integration: log-scale normalization, variable feature identification using variance stabilization transformation, data scaling, principal component analysis (PCA), shared nearest-neighbor graph construction, unsupervised clustering (resolution of 0.4), and uniform manifold approximation and projection analysis (UMAP) with 30 dimensions.

Integration of ungrafted and grafted dermis samples was performed using Seurat’s integration pipeline involving canonical correlation analysis (CCA), which corrects for batch effects. After identifying the top 2,000 highly variable features using variance stabilizing transformation, we used Seurat’s “FindIntegrationAnchors” function using 20 dimensions and 1,500 anchor features to identify anchors to integrate samples. After integration and scaling, we performed PCA using 30 principle components and 20 dimensions, UMAP analysis using 20 dimensions, and shared nearest neighbour clustering using 20 dimensions and k=50. We performed unsupervised clustering on the integrated dataset at a resolution of 0.5.

Cell clusters were annotated using canonical marker genes and top differentially expressed markers relative to other clusters, as identified using Seurat’s “FindAllMarkers” function, for which we used Wilcoxon rank sum testing with a minimum log2 fold-change of 0.1 and p-value threshold <0.05. The following cell populations were identified using the following markers: Cd8+ T cell clusters 1–3 (Cd8b1, Cd3g), myofibroblasts (Actg2, Postn), M2-polarized macrophages (Cd14, Cd16, Mrc1), arrector pili muscle cells (Acta2, Tagln, Myh11, Myl9, Mef2c, Myl4, Ppp1r14a), endothelial cells (Pecam1, Cd34), neutrophils (Cd69, Ltf), Schwann cells (Mbp), M1-polarized macrophages (Cd14, Cd16, Il1b), arginase-1+ monocytes (Cd14, Arg1), hair follicle stem cells (Krt15, Cd34, Lgr5), outer root sheath cells (Krt5, Krt6a, Krt14, Krt15, Krt16), inner root sheath cells (Krt25, Krt27, Krt28, Krt71), inner root sheath Henle’s layer (Krt25, Krt27, Krt28, Krt71++), dermal papillae clusters 1 and 2 (Vcan, Alpl, Prom1), dermal sheath/dermal cup cells (Ncam1, Itga6, Cd90, Acta2, Alpl, Itga6, Sox2, Ngfr, Cd90, Grem2), and melanocytes (Mlana, Mitf).

We then evaluated expression of all murine NKG2D ligands (Ulbp1, Ulbp2, Ulbp3, Mica, Micb, H60a, H60b, H60c, Rae1, and Mult1) in dermal mesenchymal cell populations including dermal papillae clusters 1–2 (DP1, DP2), dermal sheath/dermal cup cluster (DS/DC), outer root sheath cluster (ORS), inner root sheath Henle layer cluster (IRS, Henle), inner root sheath cluster (IRS), and melanocytes (MC). We used Seurat’s “FindAllMarkers” function with Wilcoxon rank sum testing (minimum log2FC = 0.1; p<0.05) to determine whether there was statistically significant upregulation of NKG2D ligands in specific cell clusters in AA compared to UG.

## Supplementary Files

This is a list of supplementary files associated with this preprint. Click to download.

- supplementarytables14.pdf
- supplementaryfigures.pdf

## Data Availability

Data available on request from the authors.

## Abbreviations:

AA: Alopecia Areata
BV: Blood Vasculature
DS: Dermal Sheath
GZB: Granzyme B
HF: Hair Follicle
IS: Immune Synapse
MC: Melanocytes
MHC: Major Histocompatibility Complex
MTOC: Microtubule Organizing Center
